# The experience of SSDI beneficiaries during the Medicare waiting period

**DOI:** 10.1093/geroni/igaf122.3396

**Published:** 2025-12-31

**Authors:** Sunny Kang

**Affiliations:** The University of Baltimore, Baltimore, Maryland, United States

## Abstract

This study examined the experiences of Social Security Disability Insurance (SSDI) beneficiaries during the Medicare waiting period using a mixed-methods approach. It combined semi-structured individual interviews with analyses of the National Health Interview Survey (NHIS) linked to Social Security Administration (SSA) administrative data. The Medicare waiting period represents a critical time for SSDI beneficiaries, given their poorer health and limited financial resources. In 2021, there were 572,000 new SSDI awards, with the number in the Medicare waiting period slightly lower due to deaths, recoveries, and repeat entitlements. Prior research found that before the Affordable Care Act (ACA), 25.8% of these beneficiaries lacked health insurance during this period, with male and Black beneficiaries disproportionately affected. The ACA significantly expanded insurance options, potentially altering their experiences. The quantitative component analyzed NHIS data from 2019-2024, linked to SSA’s Master Beneficiary Record. We examined health insurance coverage, healthcare access, and use among SSDI beneficiaries, applying multivariate logistic regression to assess disparities. Interaction terms evaluated moderator effects related to race, ethnicity, and income. The qualitative component involved interviews with 25 SSDI beneficiaries in Baltimore, focusing on their healthcare challenges and coping mechanisms. Thematic analysis identified key barriers to care and policy implications. Findings highlighted persistent disparities in health insurance coverage and access despite ACA-related reforms. Beneficiaries who remained uninsured reported delayed care and worsened health outcomes. This study underscores the need for policy interventions to improve coverage and healthcare access for SSDI beneficiaries during the Medicare waiting period.

